# Pursuing the objectives of support to providers and external accountability through enabling controls - a study of governance models in Swedish primary care

**DOI:** 10.1186/s12913-019-3945-0

**Published:** 2019-02-11

**Authors:** Anna Häger Glenngård

**Affiliations:** Lund University School of Economics and Management, Lund, Sweden

**Keywords:** Governance, Management control, Accountability, Coercive and enabling controls, Healthcare

## Abstract

**Background:**

The purpose of this study was to contribute to knowledge about what is regarded as an appropriate governance model in welfare markets in healthcare, from the perspective of government. The study draws on a framework about governance in healthcare systems as a continuous process of priority setting, monitoring and accountability. It relates to various dimensions of management controls; a view on management controls as a package with interdependence between different controls, a use of management controls as coercive or enabling, and implications of involving providers in the design of control systems.

**Methods:**

The empirical material is limited to experiences of governance models used in Swedish primary care. Data from the 21 county councils responsible for organizing and financing healthcare in Sweden was gathered during 2016–2017 through a survey, interviews and document review. Data was analyzed using conventional content analysis.

**Results:**

According to the county councils, governance is a continuous process. Four controls are used in all county councils: contracts, reimbursement systems, dialogue and performance measurement systems (PMS). The appropriateness of different controls is associated with their interdependence, e.g. the more formalized the use of dialogue, the more enabling the use of PMS. An appropriate governance model should on the one hand support innovations and quality improvements and on the other hand ensure external accountability for the use of allocated resources and adherence to agreements. The interviewed representatives described the intended role as both coercive and enabling but in favor of enabling. Using management controls in a way that improves the providers’ attitude towards and capacity to achieve the assigned task of delivering high-quality healthcare was described as central.

**Conclusions:**

An appropriate governance model in healthcare systems should enable governments to combine two roles: to force compliance with agreements to ensure external accountability for the use of allocated resources and to offer support to learning and quality improvement in the healthcare system. Governance can be regarded as a continuous process where several management controls operate as a package and the appropriateness of different controls is associated with their interdependence. An appropriate governance model should, from the perspective of government, encompass a high level of formalization of both coercive and enabling types of control but with greater emphasis on enabling types. Governments may pursue the objectives of support to providers and external accountability in healthcare systems by using management controls in enabling ways.

## Background

Governance can be described in terms of a continuous process consisting of three parts, namely priority setting, monitoring and accountability [1]: In publicly funded healthcare systems, governments set priorities based on population needs and overall political decisions, given system constraints (e.g. available resources) and allocate resources and tasks to healthcare providers based on such priorities. The next step for governments is to collect information and monitor provider activities. Finally, this information is used to evaluate provider activities against expectations and consequently hold providers to account through sanctions or rewards [2]. The sanctions that healthcare providers can face when their actions are not in line with the expectations range from bad reputation to being forced to stop practicing care. Similarly, the rewards if activities are in line with expectations are a good reputation and continued possibilities to provide services and get publicly reimbursed.

Governance and management are commonly regarded as the most complex but at the same time the most important functions of governments in relation to their healthcare systems [1]. The controls used by governments to monitor and hold providers to account for allocated resources and tasks can be described as a management control package [3]. Cybernetic controls, such as performance measurement systems (PMS) and reimbursement systems are commonly used. However, the different management controls do not operate in isolation but are interrelated and dependent on the context in which they are situated: Planning, cybernetic controls, rewards and compensation controls are exercised within the structure of administrative controls, such as the overall governance structure. Taking interdependence between different management controls into account is crucial not only for policy makers when designing the management control systems, but also for researchers when studying them [4]. What is regarded as an appropriate set of management controls in healthcare systems may depend on several factors. One is whose perspective you take: the government in its role as policy maker and purchaser of services (principal) or the providers who are subject to the control (agent)? Agency theory is commonly used to describe relationships between actors in healthcare systems [5]. Principals and agents may very well have different expectations and be motivated by different things. Goal alignment between different actors is often described as one of the most important tasks of management control systems [6].

What is regarded as an appropriate set of management controls can also be assumed to depend on the intended overall role of the governance model from the controlling part: To ensure external accountability for the use of allocated resources and adherence to tasks stipulated in agreements between providers and governments? Or to support learning, innovations and quality improvement in the health system? Management controls can be used in different ways and for different purposes in healthcare, e.g. to offer support to quality improvements and for external accountability [7, 8]. For example, evidence from Swedish primary care shows that county councils regard the use of results from a national patient survey (NPE) to allocate resources to healthcare providers in the context of performance-based payment (P4P) as a sharp accountability mechanism. However, to financially punish or reward providers who receive poor scores does not in itself give any support to quality improvement. If the information from the survey is to be used to support quality improvement, it needs to be used to give feed-back to providers and to be discussed in relation to other information [9, 10]. In more general terms, the management control package used can be characterized by the degree of formalization and type of controls used: coercive or enabling [11]. Degree of formalization refers to the occurrence of structured formal rules, procedures and instructions. Coercive types of control refer to procedures to force compliance while enabling types provide individuals with knowledge about lessons learned from experience. Organizations may pursue different, potentially conflicting objectives, by using managements controls in an enabling way [12]. Principals assigning governance an enabling role should invite agents to participate in the design and use of the management control systems [13, 14]. Using such a participatory process can provide agents with increased capacity to do their task through increased knowledge about the organization’s goals and how to reach them. Moreover, by involving agents, principals can improve the agents’ attitudes towards the assigned task [15].

Finally, what is regarded as an appropriate set of management controls could also depend on contextual factors: when and where. Principles for governance and management are subject to continuous change, reflecting the priorities ​​and trends that apply in the society in general. This applies not least to organizations operating in the public sector where various reforms are introduced on a regular basis as a solution to identified problems and political priorities [16]. Each new reform entails new demands on the organizations that operate here, a fact implying that governance models need to be adapted. Swedish healthcare is no exception to policy makers’ ambition to improve the healthcare system by introducing continuous reforms [17, 18]. The introduction of the choice reform in Swedish primary care in 2007–2010 implied, among other things, that the 21 county councils responsible for the organization and financing of healthcare had to change their use of management controls to fit the new governance structure [18]. The intended outcome of market reforms, including expanded citizen choice and provider competition, is that it should improve the efficiency and quality of services and the responsiveness towards citizens’ expectations through market mechanisms [19, 20]. The introduction of a welfare market implies that payment is separated from provision and private providers become involved in the delivery of public services. Then, governments allocate resources and tasks (responsibilities for collective objectives) to the public and private providers through official agreements or contracts [21]. In Swedish primary care, this overall governance structure relying on choice and competition replaced the previous traditional structure relying more on trust and altruism, which assumes that all public servants behave like knights and does not reward or punish neither success nor failure [22, 23].

In practice, a mix of governance structures is used on most contexts. Studies on Swedish primary care after the choice reform have shown that it is necessary to maintain accountability relationships between providers and governments to ensure that overall objectives of healthcare are achieved [10, 24, 25]. This applies not least in a situation with increased choice for individuals. Increased choice may suffice to achieve increased accessibility and responsiveness to individual needs and preferences, but not to achieve important goals from a population perspective. Specifying requirements that providers must comply with in order to be allowed to practice care and be publicly reimbursed, and follow-up of such requirements using performance measures of patient-reported experiences, compliance with clinical guidelines and waiting times have become common types of control following the choice reform. Hence, the overall governance structure can be described as a combination of the “choice and competition” model and the “hierarchy and targets” model where the latter is associated with external incentives and monitoring by government [22, 23]. One critique following the change in the overall governance structure is that providers are subject to a heavy administrative burden and perhaps too tight control, as they are supposed to act in accordance with evidence-based clinical guidelines, targets and clinical performance indicators set by governments aiming at greater systematization in healthcare [26, 27]. Limiting health care workers professional autonomy by the use of targets and clinical performance indicators set by governments can be described as coercive types of control [11, 26].

Overall, the Swedish healthcare system offers an interesting context for research in the area of governance and management. The central government is responsible for overall health care policy and legislation, but the responsibility for financing and organizing health care is decentralized to 21 independent county councils. The specific requirements that providers have to comply with to be allowed to practice care and the use of management controls used differ between local county councils, depending on local political considerations and priorities. This structure offers an interesting variety for research. A review from 2013 suggests that, together with contracts and reimbursement models, most county councils use some kind of dialogue combined with PMS to control providers in primary care [28]. However, there is limited knowledge about what may be regarded as an appropriate governance model from the perspective of different actors despite almost 10 years of experiences of organizing Swedish primary care in a welfare market. This study sets out to explore this topic from the perspective of governments in their role as purchaser of services. What can be regarded as an appropriate governance model? What management controls are used and what control is the most important? In what way are different controls interrelated? Is the intended role of governance to force compliance with agreements to ensure external accountability for the use of allocated resources or to offer support to learning and quality improvement?

### Purpose

The purpose of this study is to contribute to knowledge about what is regarded as an appropriate governance model in welfare markets in healthcare from the perspective of government. It is based on experiences of management controls used by county councils in Swedish primary care.

## Methods

### Context of the study

Swedish primary care is organized in a regulated market (welfare market) with freedom of choice for individuals and competition between providers. There are about 1200 primary care practices in Sweden, of which about 40% are privately operated (Table 1). A clear majority of private primary care providers are for profit. Primary care accounts for about one fifth of the total healthcare expenditures in Sweden. The share varies slightly between county councils. Team-based primary care facilities with different staff categories (GPs, nurses, midwives, physiotherapists and psychologists) is the most common form of primary care practice.

**Table 1:** Number of primary care practices, and proportion of private practices in all county councils year 2016/17

County council	All	Private	Private (%)
Blekinge	19	7	37%
Dalarna	28	5	18%
Gotland	7	2	29%
Gävleborg	43	16	37%
Halland	48	24	50%
Jönköping	46	15	33%
Kalmar	37	10	27%
Kronoberg	32	11	34%
Landstinget i Värmland	37	9	24%
Norrbotten	34	4	12%
Region Jämtland Härjedalen	28	4	14%
Skåne from 2015	150	75	50%
Stockholm from 2016	207	140	68%
Södermanland	27	9	33%
Uppsala	52	26	50%
VGR	200	80	40%
Västerbotten	39	7	18%
Västernorrland	32	12	38%
Västmanland	27	16	59%
Örebro	29	4	14%
Östergötland	42	9	21%
Sweden	1164	485	42%

County councils, in their role as purchasers, use contracts to allocate resources and tasks to healthcare providers. The contracts are based on financial, organisational and quality requirements that providers must comply with to be allowed to practice primary care are and get publicly reimbursed. It is up to each county council to decide on the specific requirements but the same requirements apply to private and public providers (Healthcare Act SFS 1982: 763 5§; Act 2009: 140). The county councils approve providers who meet the specified requirements and then sign a contract that shows that the provider is allowed to practice primary care and get publicly reimbursed. These contracts are signed between the contract manager in each county council and the managing director at each primary care practice.

### Operationalization of conceptual framework

The conceptual frame of reference presented in the background section is operationalized and adapted to the context of the study in the following way:

- The relationship studied is that between county councils and primary care providers, where the former is the purchaser of services (i.e. principal) and the latter is the provider of services (i.e. agent).
- Governance models refer to the set of management controls, i.e. the management control system used by county councils to control primary care providers.
- The concept of “appropriate governance model” refers to views held by county councils in their role as purchaser of services.
- Coercive controls refer to management controls that are designed to ensure external accountability for the use of allocated resources and adherence to tasks stipulated in contracts between providers and county councils.
- Enabling controls refer to management controls that are designed to support learning and quality improvements.

### Collection of empirical material

#### Sample

All 21 county councils were invited to participate in the study by answering a survey and participating in a telephone interview. Contact information to representatives with documented knowledge about governance and management in primary care in each county council was obtained from the Swedish Association for Local Authorities and Regions, SALAR.

#### Survey

A survey was sent by e-mail to all representatives in February 2016 and reminders were sent in March, April and May. They were also able to answer the survey during the subsequent telephone interview. Eleven representatives completed the survey before the interviews and additional eight during the subsequent interviews, i.e. survey data was obtained from 19 out of 21 county councils in total (see Table 2).

**Table 2:** Use of PMS in Swedish primary care year 2016/17 according to survey and interviews with county council representatives (*N* = 19 respondents)

	Used for monitoring?	Number of indicators	Used in P4P?
Blekinge	Yes	Four areas	Yes (4 targets)
Dalarna	Yes	Approx. 30	Yes (3 targets)
Gävleborg	Yes	Approx. 30	Yes (3 targets)
Halland	Yes	Approx. 80	Yes (1 target)
Jönköping	Yes	Approx. 80	Yes (7 targets)
Kalmar	Yes	Approx. 15	No
Kronoberg	Yes	Approx. 25	Yes (14 targets including coverage rate)
Landstinget i Värmland	Yes	Approx. 80	No
Norrbotten	Yes	Approx. 40	Yes (1 target)
Region Jämtland Härjedalen	Yes	Approx. 50	No
Skåne*	Yes	Approx. 80	Yes (coverage rate)
Stockholm	Yes	Approx. 80	Yes (3 targets)**
Södermanland	Yes	Approx. 70	Yes (accessibility)
Uppsala	Yes	Approx. 80	Yes (7 targets)
VGR	Yes	Approx. 80	Yes (9 targets including coverage rate)
Västerbotten	Yes	Approx. 40	Yes (6 targets)
Västernorrland	Yes	Approx. 40	No (not efter 2016)
Örebro	Yes	Approx. 65	Yes (7 targets)
Östergötland	Yes	Four areas	No

^*^15 measures were monitored whereof five were used in P4P Schemes 2009–2014

^**^15 measures were used in P4P Schemes 2009–2015

The purpose of the survey was to get an overall picture of the development, use and experiences of different management controls in Swedish primary care. The survey results are reported in full elsewhere [29, 30]. This study is limited to an analysis of 15 questions:

- 10 open questions asking the respondent to describe the development and use of management controls, including open questions about requirements that providers must comply with, and the use and role of dialogue, PMS and reimbursement systems.
- 5 questions using a 7-point Likert scale asking the respondents to rate the occurrence, scope and view on different controls: To what extent do you use dialogue (1 = Very small extent, 7 = Very high extent)? To what extent do you use PMS (1 = Very small extent, 7 = Very high extent)? Is the role of dialogue mainly to support learning and quality improvements or mainly to ensure external accountability for the use of allocated resources and adherence to agreements (1 = mainly support, 4 = equally support and accountability, 7 = mainly accountability)? Is the role of PMS mainly to support learning and quality improvements or mainly to ensure external accountability for the use of allocated resources and adherence to agreements (1 = mainly support, 4 = equally support and accountability, 7 = mainly accountability)? Is the role of the overall governance model mainly to support learning and quality improvements or mainly to ensure external accountability for the use of allocated resources and adherence to agreements (1 = mainly support, 4 = equally support and accountability, 7 = mainly accountability)?

#### Interviews

Semi-structured telephone interviews were conducted with representatives from 19 county councils in March–June 2016. The interviews lasted between 30 and 75 min. In the cases where the survey had not been answered in advance, the interviews were generally longer as the survey questions were answered before the actual interview started. The interviews were recorded but not transcribed. The purpose was to gain additional and a more in-depth knowledge about the development, use and experiences of different management controls and views about their appropriateness and interrelationships. Results from the interviews are reported in full elsewhere [29, 30]. This study is limited to an analysis of the following areas covered in each interview:

- The survey showed that different concepts (e.g. clinical audit, dialogue and PMS) were used in different ways. Therefore the first part of the interviews focused on ensuring a mutual understanding of concepts related to management controls to avoid misunderstandings. This also led to a modification of the survey responses in some cases.
- One area was about describing the use of different management controls and to reflect upon the most important part of the governance model.
- One area concerned the interviewee’s reflections upon the appropriateness, role and interrelationships of different controls used as well as the governance model as a whole. This area of reflection included his or her view on the role of controls to support learning and quality improvements and to ensure external accountability for the use of allocated resources and adherence to agreements.
- One area was about providing concrete examples of if and how different controls help ensuring external accountability for the use of allocated resources and adherence to tasks stipulated in agreements between providers and county councils and support learning and quality improvements, respectively.

#### Document review

Data was also collected through a review of tender documents, retrieved from the websites of all 21 county councils. The tender document specifies the financial, organisational and quality requirements that providers must comply with to be allowed to practice primary care and be publicly reimbursed in each county council. The tender document underlies the contracts signed between the contract manager in each county council and the managing director at each primary care practice. The review was used to support findings from the interviews regarding the scope of services that providers are supposed to deliver and principles for paying primary care providers in different county councils. A comparative analysis of the contents of the tender documents is provided elsewhere [29, 30].

#### Analysis and validation of results

Conventional content analysis was used to analyze the collected data [31]. As a starting point, the survey results, interviews and tender documents were reviewed to get an overall picture of the empirical findings. Thereafter a more thorough analysis, focusing on categorizing and describing the material was done. Three categories were used based on the conceptual framework (category 1 and 2) and the initial review (category 3): 1) The use, importance and interrelations of different management controls; 2) The intended role of different controls and the governance model as a whole according to county councils in their role as purchasers of health care, 3) The use of management controls to support innovations and quality improvements. As far as possible, data from the survey, interviews and tender documents have been used to triangulate findings. However, interview data has been given priority in case findings from the survey and the interview diverged. Quotes are used to illustrate the empirical results as far as possible.

Preliminary empirical findings were presented (approx. 30 min) at a workshop organized by SALAR in October 2016, where individuals working with the development and use of governance and management models in primary care in all county councils participated. Following the presentation, there was a general discussion about the findings (approx. 30 min). Following the workshop, the empirical material was slightly revised and then sent to all interviewed county council representatives for validation in February 2017. 12 came back with minor changes over phone or e-mail. The revised empirical material and preliminary conclusions, based on an analysis of the empirical findings through the lens of the conceptual framework, was presented and discussed at a seminar in April 2017, where the participants represented both practitioners and researchers with an interest in public sector governance and management. The discussion provided useful insights and guidance to the discussion of the empirical results and the conclusions drawn. Since the study covers data from 21 cases, it is necessary to generalize when analyzing and presenting the results and conclusions, however.

## Results

### Governance models in Swedish primary care

The county council representatives described their governance model in primary care in terms of a continuous process and a package consisting several management controls. They said that an appropriate governance model should ensure that providers deliver high-quality care to all citizens.

### The use and importance of different management controls

When asked about the use of management controls, four controls were identified in all county councils: contracts, reimbursement systems, dialogue and performance measurement systems (PMS). When asked the question: “What is the most important component of your governance model in primary care?” the most common answers were along the line: “*There is no one most important component in the governance model. It is a process, an ongoing work with monitoring of provider activities, learning what works well and less well and then adjusting the tender document accordingly*” or “*You cannot pick one component. It is not one part but a package with many parts. This includes the tender document and contract and then monitoring that this is followed using both performance measures and dialogue*” or both. Rewards and sanctions were also mentioned on an overall level: Rewards to providers fulfilling their assigned task range from positive feed-back, good reputation (through transparent presentation of performance measures and/or sharing of results from successful working methods) to financial rewards (through primarily P4P schemes). Sanctions for providers not fulfilling their assigned task range from negative feed-back at dialogue meetings, bad reputation (through transparent presentation of performance measures), warnings and withheld payment, to ultimately forcing providers to stop practicing primary care.

When specifically asked to identify the most important component, the most common response was dialogue and the tender document underlying contracts between purchasers and providers, followed by PMS and the reimbursement system. The overall impression from the interviews was that the county council representatives considered dialogue to be a more appropriate type of control than PMS, as it builds trust, relationships and shared knowledge between purchasers and providers. Two quotes illustrate this observation:

*“We visit all providers and have a dialogue. When we ask for results, something happens in the organization. ... It’s difficult to control behavior using money. … payment often misses the point.” (Dalarna).*

*“Continuity in relations and a dialogue between purchasers and providers and to be consistent over time. The objectives in primary care should not only be decided by politicians, they should be developed in dialogue with healthcare providers. This is crucial for a sustainable primary care.” (Gävleborg).*

The county council representatives described the relations between themselves and providers resulting from regular dialogue as the core of an appropriate governance model, to build trust between purchasers and provider and to improve the usefulness of PMS:

*“We try to help providers develop their quality. ... We monitor about 70 indicators. Here we see what stands out. ... control can be done with indicators. Dialogue is needed primarily to develop the quality of care, in order to make the outcomes of indicators useful. “(Sörmland).*

*“The dialogue-based parts aim at both support and control. You can be sharp also when you have good relationships. “(Västernorrland).*

Although the relations between purchasers and providers resulting from regular dialogue was described as the core of an appropriate governance model, some county council representatives expressed a view that the provider reimbursement system in fact is a more powerful control system:

*“Dialogue ideally but the reimbursement system is crucial for providers” (Kalmar).*

The scope of services that providers should deliver and the design of the reimbursement system vary between the county councils depending on local priorities and traditions. A review of the tender documents shows that the scope of services is rather broad, however. Providers are assigned a comprehensive responsibility for outpatient care among enrolled individuals. Regarding reimbursement systems, a combination of fixed capitation for enrolled individuals and variable payment for visits is used in all county councils. The share of the fixed capitation varies between 60% (Stockholm) and almost 100% (Jämtland-Härjedalen, Värmland, Halland, Västernorrland) of total payment. The fixed capitation is adjusted for age, overall illness (ACG) and socio-economic conditions (CNI) among enrolled individuals in a majority of cases. Performance-based payment (P4P) is used in two thirds of all county council (Table 2). In two cases (Kalmar and Gävleborg) there is also a small proportion of funds earmarked for work with innovations and quality improvements (in addition to P4P) at each primary care practice. A comparative analysis of the reimbursement models and the scope of services specified in the tender documents is provided elsewhere [29, 30].

### PMS and dialogue are used and interrelated to a high extent

**Fig. 1:**
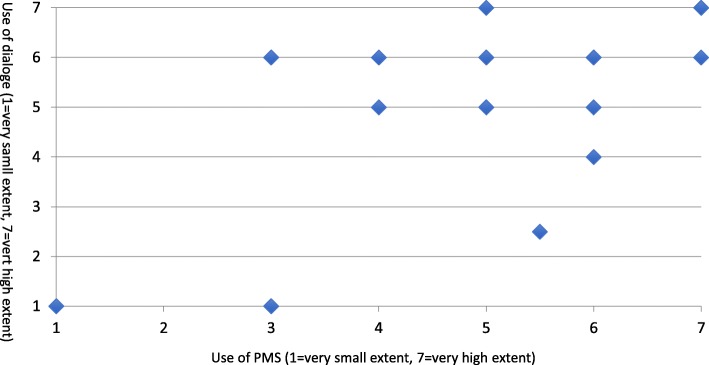
Use of dialogue^a^ and PMS^b^

The results from the survey and the interviews show that both PMS and dialogue is used in most county councils and that the appropriateness of these two controls are associated with their interdependence. Generally, the extent to which county councils use both dialogue and PMS is high according to the results from the survey (Fig. 1).

When asked to describe the use of PMS, all county council representatives explained that they use this type of control to monitor healthcare providers. The number of indicators continuously monitored varies between about 15 and about 80. The indicators are in most cases structure and process measures which assess care provided to different patient groups, such as continuity and accessibility, if there is a diabetes nurse at the primary care practice, the proportion of newborns who receive home visits, work with drug reviews among elderly patients or compliance with clinical guidelines regarding prescription of antibiotics and/or other drugs. In a few cases, outcome measures are used, such as avoidable hospitalization rates among elderly and patient reported outcome measures (PREM). Several county councils use the approximately 80 indicators that have been developed within the framework of Primary Care Quality, a national collaboration between a number of county councils led by SALAR. In two thirds of the county councils the outcome in indicators are used to allocate resources to providers in pay-for-performance schemes (P4P).

The county council representatives explained that the use of PMS has evolved over time. Initially, they focused solely on monitoring volumes of activities with the aim of measuring productivity. Gradually, PMS has also become valuable as a control to offer support to the development of working methods and quality improvements linked to the specific conditions facing each provider. County councils give formalized feedback during dialogue meetings with individual providers but also in groups where common problems and solutions to these are raised and discussed by several providers together. Many county councils publish results and make them available to providers to enable comparison or results with each other – a form of benchmarking.

According to the survey, 17 of the 19 county councils who participated in the study engage in regular dialogue meetings with all providers. Thirteen meet with each provider individually or groups of providers, or both, while four only meet with groups of providers. The representatives from the county councils explained that the content of the dialogue meetings has shifted over time. When different forms of dialogue were introduced in connection with the choice reform, i.e. around 2010, the scope was more about accountability: The initial focus was on explaining and clarifying the requirements for accreditation and payment, following up activities and improving ways of measuring quality in primary care. Gradually this type of control has become more to offer support to learning and quality improvements: The content has shifted towards discussions about working methods with respect to the needs of the patient population at each practice and feedback on outcome in performance measures.

Several county council representatives stressed the importance of dialogue to build strong purchaser-provider relations and trust between themselves and the providers but also to enable appropriate monitoring of activities. They also explained that good relations can improve the attitude towards the assigned task among providers:

*“Human relations are crucial in healthcare. You cannot measure and follow up care without face-to-face meetings. It can also motivate providers to implement changes if needed.” (Kalmar).*

Another benefit with dialogue, according to the county council representatives, is to create a mutual understanding of the actual task assignment to providers in contracts. Dialogue meetings play an important role in explaining and translating what is stated in the tender documents that underlie the contracts between county councils and providers. This creates an understanding and increases the capacity among providers to fulfill their task. Moreover, when county councils take part in dialogue with providers this creates an understanding of what works and does not work in practice. They use such information when revising the tender document in order to align the task assigned to providers with what is actually possible to achieve in the day-to-day work.

### The intended role of governance and management controls

**Fig. 2:**
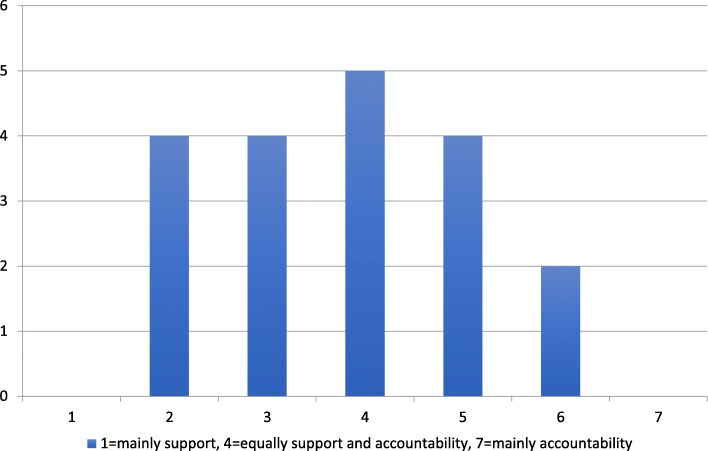
The role of governance as enabling or coercive

When asked the question “Is the overall role of governance in primary care mainly to offer support to learning and quality improvements or mainly to ensure external accountability for the use of allocated resources and adherence to agreements?”, most county council representatives responded that it was both. They said that it is necessary to combine both roles to ensure that providers deliver high-quality care to all citizens. The answers from the survey also indicate that the intended role of governance is both coercive and enabling in most cases with a slight tilt towards enabling (Fig. 2).

According to the results from the survey, there is a systematic difference in the views about the role of the overall governance model and the overall occurrence of structured formal procedures regarding the use of PMS and dialogue. Characteristics of the governance models where the respondents stated that the role of governance is equally to offer support and ensure accountability is a large occurrence of structured formal procedures. This refers to e.g. procedures for reporting and giving feed-back on measures and structures for regular dialogue. In county councils where role of governance was described as equally to offer support and ensure accountability both controls are used approximately to the same extent. Among those who stated that the role of governance is primarily to offer support, PMS and dialogue is also used in a structured formal way to a large extent. Dialogue is used to greater extent than PMS however. Among those who responded that the role of governance is primarily to ensure accountability, the overall occurrence of structured formal procedures regarding the use of primarily dialogue is lower. In that group, PMS is used to a larger extent than dialogue (Table 3).

**Table 3:** Support and formalization go hand in hand

	Primarily support*	Equally support and accountability**	Primarily accountability***
Overall formalized use of dialogue and PMS	Both dialogue and PMS are used to a higher extent	Both dialogue and PMS are used to a higher extent	Both dialogue and PMS are used to a lower extent
Balance between PMS and dialogue	More use of dialogue than PMS	Equal use of dialogue and PMS	More use of PMS than dialogue

^*^1–3 on the question “Is the role of the overall governance model mainly to support learning and quality improvements or mainly to ensure external accountability for the use of allocated resources and adherence to agreements?” (where 1 = mainly support, 4 = equally support and accountability, 7 = mainly accountability). *N* = 8 county councils

^**^4 on the question “Is the role of the overall governance model mainly to support learning and quality improvements or mainly to ensure external accountability for the use of allocated resources and adherence to agreements?” (where 1 = mainly support, 4 = equally support and accountability, 7 = mainly accountability). *N* = 6 county councils

^***^5–7 on the question “Is the role of the overall governance model mainly to support learning and quality improvements or mainly to ensure external accountability for the use of allocated resources and adherence to agreements?” (where 1 = mainly support, 4 = equally support and accountability, 7 = mainly accountability). *N* = 7 county councils

### Use of management controls to promote innovations and quality improvements

Although it was not a specific area in the survey or the interview guide, all county council representatives explained that an appropriate governance model should offer support to work with quality improvements and innovative solutions to identified problems and challenges. This is regarded as crucial to achieve high-quality care to all citizens. Different management controls are used for this purpose.

First of all, county councils use dialogue to offer support to quality improvements and continuous learning. Providers get support to improve their services and to be more responsive to their patients through feedback and discussions about outcomes in different indicators (over time and in comparison with others) taking into account the specific conditions for each provider. Moreover, the respondents described regular dialogue as a way to learn from successful experiences about how individual providers have used innovative solutions to tackle different problems or difficulties. County councils share lessons from such “good examples” with other providers at conferences and regular dialogue meetings to support continuous learning and quality improvements among providers. Two examples brought up are ways to direct patients to the best level of care through the use of a well-functioning triage model and strategies to increase the coverage of seasonal vaccine against influenza. A third example regards the coordination of care for palliative patients.

Secondly, county councils use PMS to promote quality developments. All county councils work with follow-up of quality indicators in primary care in 2016/17 (see Table 2). PMS is used to support quality development work through feedback to providers at dialogue meetings. Providers´ performance according to different quality indicators is analyzed over time and in comparison with other providers and in relation to the case mix and other conditions facing each provider. Benchmarking, whereby the outcome in quality indicators is publicly disseminated and compared between providers, is also used to promote continuous quality improvements. Nobody wants to be the worst! One example regards diabetes care:

*“We have previously been among the worst in Sweden when it comes to care for patients with type 2 diabetes. A development project has been carried out with visits to all primary care practices. We have based dialogue meetings on indicators and comparisons of results and the quality has improved. We believe that targets and PMS motivate improvements - to make it better for patients. The development work in diabetes started in 2005 and has had a long-term focus with clear goals and feedback of results.” (Örebro).*

Third, county councils use the reimbursement system to support innovations and quality improvements. In 14 of the 21 county councils, P4P is used to promote quality improvements in 2016/17. The most common measures are process indicators, focusing on accessibility, coverage rate, prevention and compliance with various clinical guidelines. One county council uses a comprehensive assessment of the outcome in all indicators instead of focusing on individual measures, and then appoints the provider of the year:

*“We have developed an evaluation model in which we assess all data available, for example NPE [National Patient Survey], accessibility, hygiene standards and antibiotics. The provider that improves the most compared to the previous year is rewarded with a fixed sum to be used for development work the coming year.” (Jämtland-Härjedalen).*

In two cases (Kalmar and Gävleborg), funds earmarked for development work is allocated to healthcare practices prospectively and the providers are trusted to use the funds as they see fit. Dialogue is used to follow up the use of these earmarked funds. Moreover, all development work is published and shared with other providers to support quality improvement:

*“Since 2015, all primary care practices are allocated funds for development work prospectively. The only restriction is that the results should be shared. The practice with the most interesting development work presents their experiences at special workshops. The intention is that healthcare staff should be able to share experiences and listen and get inspired. We publish all presentations on our website. Improvement work may, for example, be the way providers work with mental illness and acute care, but also information for asylum seekers.” (Gävleborg).*

## Discussion

This study contributes to knowledge about what is regarded as an appropriate governance model in welfare markets in healthcare from the perspective of government. An important limitation thus is that it focuses on the perspective of the principal rather than the agent. It is based on data from the 21 county councils responsible for organizing and financing healthcare in Sweden, against the background of almost 10 years of experiences of governance and management in an overall governance structure based on a combination of the “choice and competition” model and the “hierarchy and targets” model [22, 23].

### Governance as a continuous process and a management control package

The county council representatives explained that the overall role of governance in Swedish primary care is to ensure that providers deliver high-quality care to all citizens. This view can be interpreted as a combination of the two roles suggested in the conceptual framework of this paper: to force compliance with agreements to ensure external accountability for the use of allocated resources and to offer support to learning and quality improvement in the health system. Hence, what is regarded as an appropriate governance model from the perspective of governments, in their role as purchaser of health care, is a set of management controls enabling them to pursue these two objectives simultaneously. This stance is reflected by a view on governance models as a continuous process and a package of management controls, where different controls complement each other.

The results show that the view on governance models in Swedish primary care are very much in line with the description of governance in healthcare system as a continuous process with three parts [1]. The financial, organisational and quality requirements that providers must comply with to be allowed to practice primary and be publicly reimbursed reflect the local priorities and are formalized in the tender documents which then forms the basis for contracts between county councils and providers. Dialogue and PMS are used to monitor provider activities. County councils also continuously revise the tender documents based on shared knowledge about providers capacity to accomplish their desired task, resulting from dialogue meetings. This also improves the ability for providers to carry out the task assigned to them. Finally, providers fulfilling their assigned task are rewarded through e.g. positive feed-back and a good reputation while providers not fulfilling their assigned task are sanctioned through e.g. warnings and withheld payment.

The view on governance models in Swedish primary care as a combination of several interdependent management controls is also very much in line with the definition of management control as a package [3]. Malmi and Brown (2008) suggest that there is a need to complement survey questionnaires with interview data to generate further understanding of how different management controls operate as a package. This approach was adopted in the current study to generate more understanding about the use, appropriateness and interplay of different controls used by government in Swedish primary care. Administrative controls (contracts and dialogue) were described as the most central object in all cases: The tender document specifies the requirements that providers are expected to fulfill in order to be allowed to practice primary care and be publicly reimbursed in each county. The contracts between providers and county councils are based on this document. Other central parts can be sorted under cybernetic controls: PMS and reimbursement systems were also described as central. From the perspective of the county councils, both PMS and dialogue are needed as they complement and improve each other. All county councils use a combination of dialogue and PMS to support learning and quality improvements and to ensure external accountability for the use of allocated resources and adherence to agreements.

### The intended role of governance is both enabling and coercive

In terms of the framework by Adler and Borys (1996) all county council representatives explained that the intended role of the governance models is both coercive and enabling. Dialogue and PMS are treated as complementary management controls. Dialogue is considered to be a somewhat more enabling type of control than PMS but only marginally at the overall level, according to the results in this study. However, the more formalized the use of dialogue, the more enabling the use of PMS is perceived to be according to the interviewed county council representatives. The identified interdependence between PMS and dialogue and between contracts and dialogue in this study furthers the findings in previous research. Similar to Grabner and Moers (2013), the results demonstrate that the use of one management control practice may improve the appropriateness and usefulness of another practice. This interdependence can, in turn, be linked to the elements of participatory process [15] associated with the use of dialogue when developing the PMS and revising the tender documents [14]. Involving those who are subject to the control in the design of the control system enhances both capabilities to perform the assigned task and a positive attitude towards the controlling part according to previous research [15]. It can also be linked to the way in which feed-back is given to providers. In structured dialogue meetings, the outcome of different indicators can be put into a relevant context and constitute a basis for a discussion about how to improve working methods and the quality of care. In this way, PMS is also used to support learning and quality improvements. Hence, the results support the arguments in previous research in Sweden [9] and elsewhere [7, 8] that PMS can be used in different ways. From a more practical policy perspective, the result about the interplay between PMS and dialogue is interesting in light of the arguments brought forward by medical professionals in Sweden that they are subject to a heavy administrative burden, related to PMS designed by governments [26, 27]. An increased use of dialogue as a forum to inform the design PMS and to give providers feed-back might improve the perceived usefulness of PMS among healthcare professionals, i.e. improve the attitude towards the controlling part [15].

From the perspective of government, using management controls in an enabling way also fosters good purchaser-provider relations and trust between providers and the controlling part. The county council representatives explained that good purchaser-provider relations, in turn, are key to foster change. The use of the reimbursement system to support innovations and quality improvements in Gävleborg illustrates a fruitful combination of formalized enabling controls, trust and innovations: In this case, the county council allocates funds for development work to primary care practices prospectively and providers are trusted to use funds as they see fit. At the same time, there is a high degree of formalization in the monitoring and accountability of the use of these funds, where each practice is to share its experiences for others to learn and get inspired.

### What is an appropriate governance model?

Previous research show that managers can pursue the objectives of efficiency and flexibility in service organizations by using management controls in enabling ways [12]. In a similar vein, the results in this study suggest that governments can pursue the objectives of support to providers and external accountability by using management controls in enabling ways. An appropriate governance model in welfare markets in healthcare should, from the perspective of government, encompass a high level of formalization of both coercive and enabling types of control but with greater emphasis on enabling types. This conclusion should be considered with regard to the perceptions about the overall role of governance in the studied context: to ensure that providers deliver high-quality care to all citizens. The use of management controls that Swedish county councils consider to be appropriate has great resemblance to what can be described as enabling bureaucracy [11]. The concept of enabling bureaucracy was developed by Adler and Borys in the early 1990s to describe relations between managers and employees in the context of manufacturing companies in the US. The results in this study suggest that the concept is valid also to describe relations between purchasers and providers in publicly funded health care systems in the late 2010s, at least from the government perspective. E.g. Adler and Borys find that frequent reality checks are necessary to encourage enabling formalization, one reason being to overcome information asymmetries between managers and employees. This finding is applicable also to the current study where county councils perceived the use of regular dialogue as crucial for appropriate monitoring of services and mutual learning and to foster strong purchaser-provider relations between themselves and the providers.

What is regarded as an appropriate governance model can be assumed to vary with regard to several things. I will end this paper by discussing the generalizability of results with respect to two factors raised in the background: To whom is the governance model regarded as appropriate? When and where is the governance model regarded as appropriate?

### Who?

Perceptions about an appropriate governance model in welfare markets in healthcare may vary depending on who you ask. This study reflects the principals’ perception. From the perspective of government, the use of management controls to pursue the objectives of offering support to providers to continuously improve their quality and ensuring external accountability for assigned tasks, based on priorities from a population perspective, seems crucial. However, from the perspective of providers, in a welfare market with choice for individuals and competition among providers, responsiveness to individual patients with different needs and preferences is also crucial. The ability to support healthcare providers in balancing requirements and preferences from both individuals and governments is an interesting area for further research.

The results suggest that governments emphasize the importance of trust between purchasers and providers in order to continuously develop the quality and effectiveness of the care offered to citizens. Thoughts on how such trust can be created and maintained were also raised during the interviews. One common view was that trust is dependent on good relations, which require meetings and dialogue. Hence, trust is not about absence of control. From the perspective of the principal, governance models reflecting trust involves a large use of dialogue but potentially also a large use of PMS. In order for PMS to be regarded as an enabling type of control, the indicators used should be carefully selected by purchasers and provider together. In the view of the county council representatives an understanding about what is possible to achieve in the day-to-day work and which indicators that actually reflect the quality of care is important for PMS to be perceived as meaningful for providers in their work. Furthermore, feedback to providers about the outcome and development in different indicators is crucial for PMS to support quality improvements and innovative working methods.

This study does not reflect healthcare providers’ (agents) views about governance and management. Previous research indicates that views about the role of governance, as described by the county council representatives in this study, at least partly could be similar among providers. More visible control had positive effects in 120 non-profit organisations, funded by the Swedish donor agency Sida, according to a previous study [32]. Goal-fulfilment, both observed and as perceived by managers, improved as managers experienced the control in itself to be encouraging as their performance became visible to someone outside the organisation. Increased formalization, therefore, does not necessarily need to have negative consequences for the professionally driven motivation. It can rather be linked to the type of control and in particular the way in which feedback is given to providers. In a recent study commissioned by the Swedish Trust delegation [33], the views about governance and management, with particular focus on reimbursement systems among primary care providers in two Swedish county councils, were analyzed. The results show that providers value dialogue with the principal. Dialogue was considered to be central in creating a high level of trust. These studies indicate that perceptions about what is regarded as an appropriate governance model at least partly are similar between governments and providers. Nevertheless, perceptions about the role of governance among providers and its relation to innovation and trust is another interesting area for further research.

### When and where?

Governance models are continuously changing as changes in healthcare systems occur. The triangle of equity, efficiency and cost is often seen as an overarching bane of health policy analysts [34]. There is no simple solution to how to best organize services to increase efficiency and quality without adverse consequences in terms of increased inequalities. Views about governance and management control likely reflect the priorities ​​and trends that apply in the society in general. The empirical material in this study is limited to experiences of governance models used in Swedish primary care 2016/17. Given this constraint, there seems to be a general belief that governance models characterized by enabling controls is superior to models characterized by coercive controls and that dialogue is superior to PMS and reimbursement systems. Moreover, concepts like trust and innovations were mentioned by most county council representatives. One topic for debate after the introduction of choice and competition in Swedish primary care is that governance to a large extent has come to focus on PMS. The critique against monitoring of indicators has been extensive, especially from the medical professionals. Do we measure what is relevant or what is easy to measure? Do healthcare providers have time to take care of patients or are they overloaded with administrative work? Perhaps the use of PMS is not the best way to support quality improvements and foster innovations but rather a type of control for governments aiming at greater systematization in healthcare [26]? There is also a discussion about how governance and management, including reimbursement systems, can promote innovation and trust, partly linked to the Trust delegation that was appointed by the government in 2016 [33, 35]. The Trust delegation is to provide answers to questions such as: How can governance in the public sector contribute to quality developments and innovations? With the trust reform, the government wants to develop governance models based on trust in the public sector, where professional knowledge is in focus in order to create greater benefits and quality for citizens using publicly funded services. These kinds of discussions could have influenced the findings in favor of dialogue, enabling controls and trust. To what extent the conclusions about what is regarded an appropriate governance model in welfare markets in healthcare are valid over time and in other contexts is a third interesting topic for further research.

## Conclusions

Based on experiences from Swedish primary care, this study contributes to knowledge about what is regarded as an appropriate governance model in welfare markets in healthcare from the perspective of government, in its role as purchaser. The results suggest that an appropriate governance model should enable governments to combine two roles: to force compliance with agreements to ensure external accountability for the use of allocated resources and to offer support to learning and quality improvement in the healthcare system. The results further suggest that governance in healthcare systems can be regarded as a continuous process [1] where several management controls operate as a package and the appropriateness of different controls is associated with their interdependence [3, 4]. In conclusion, an appropriate governance model in healthcare systems should encompass a high level of formalization of both coercive and enabling types of control [11] but with greater emphasis on enabling types. Governments may pursue the objectives of support to providers and external accountability by using management controls in enabling ways. A next step would be to investigate what providers consider to be an appropriate governance model. As the empirical material is limited to experiences of governance models used in Swedish primary care 2016/17 a further step would be to investigate to what extent the conclusions are valid over time and in other contexts characterized by other trends and priorities.
